# Temperature-dependent photoluminescence of surface-engineered silicon nanocrystals

**DOI:** 10.1038/srep27727

**Published:** 2016-06-14

**Authors:** Somak Mitra, Vladimir Švrček, Manual Macias-Montero, Tamilselvan Velusamy, Davide Mariotti

**Affiliations:** 1 Nanotechnology & Integrated Bio-Engineering Centre-NIBEC, Ulster University, UK; 2 Research Center for Photovoltaic Technologies, AIST, Tsukuba, 305-8568, Japan

## Abstract

In this work we report on temperature-dependent photoluminescence measurements (15–300 K), which have allowed probing radiative transitions and understanding of the appearance of various transitions. We further demonstrate that transitions associated with oxide in SiNCs show characteristic vibronic peaks that vary with surface characteristics. In particular we study differences and similarities between silicon nanocrystals (SiNCs) derived from porous silicon and SiNCs that were surface-treated using a radio-frequency (RF) microplasma system.

Silicon nanostructures received great attention due to their visible luminescence and have been considered for opto-electronic and bio–medical applications^1^,^2^. The use of silicon in a range of devices can present advantages over other elements, due to silicon abundance, non–toxicity, limited environmental footprint and well-established Si–based technologies. In the past two decades, interesting progress has been made to understand and optimize light emission from quantum confined silicon nanocrystals (SiNCs), which are crystalline nanoparticles with less than ~10 nm diameter^1^,^2^,^3^. The origin of the photoluminescence (PL) from SiNCs has been largely debated and it has become a consensus that quantum confinement models should be used in order to describe appropriately light emission^3^. However, the indirect nature of silicon has complicated the development of accurate models that depart from the theoretical description used for direct bandgap quantum confined materials^3^. Moreover, SiNCs exhibit highly reactive surfaces and present further aspects that can drastically influence optical and electrical properties^3^,^4^,^5^,^6^. Although the core of the SiNCs can be defect-free, it is clear that defect-free surface states still present many challenges that have not been resolved by existing synthesis routes^3^,^7^.

Specifically, for SiNCs derived from porous silicon, the PL peak intensity and the peak wavelength are sensitive to the surface characteristics and depend, for instance, on the extent of oxygen vs. hydrogen terminations. Most frequently, SiNCs are produced with H-terminations, however they are prone to oxidation, in particular when exposed to water vapour and in colloids^8^. Uncontrolled oxidation is detrimental to the stability of colloidal SiNCs (e.g. in ethanol or water) as the PL intensity drastically decreases over time^6^. The direct link between surface and optical properties is understandable in terms of the wavefunction in the NCs which covers the volume of the NCs and also includes the surface; this can result in changes to the density of states and as a consequence to the bandgap. Wolkin *et al*.^9^ showed that oxidation at the surface of the SiNCs can create surface localized states which may facilitate recombination process. Carriers in the NCs can diffuse from the core to the surface or vice-verse affecting the transition dynamics. One of the main remaining challenges is therefore the ability of controlling carrier’s life-time and recombination processes so that desired opto–electronic properties can be achieved. Surface engineering approaches that can provide different and new transition dynamics are therefore desirable^3^,^8^,^10^,^11^.

In this work, we therefore report on the temperature-dependent photoluminescence of un-passivated SiNCs as well as passivated SiNCs. These results have provided unique information and insight on carriers and transitions behaviors. Temperature-dependent measurements allow the possibility of reducing/enhancing given transitions and therefore isolating the contribution of specific carrier population to the photoluminescent emission. We have performed the analysis for both SiNCs derived from porous silicon and as well as surface engineered SiNCs. In order to modify the surface characteristics of un-passivated SiNCs, we have used a surface engineering technique that takes advantage of non–equilibrium and kinetically driven liquid chemistries initiated by atmospheric pressure plasmas.

## Methods

**Figure 1:**
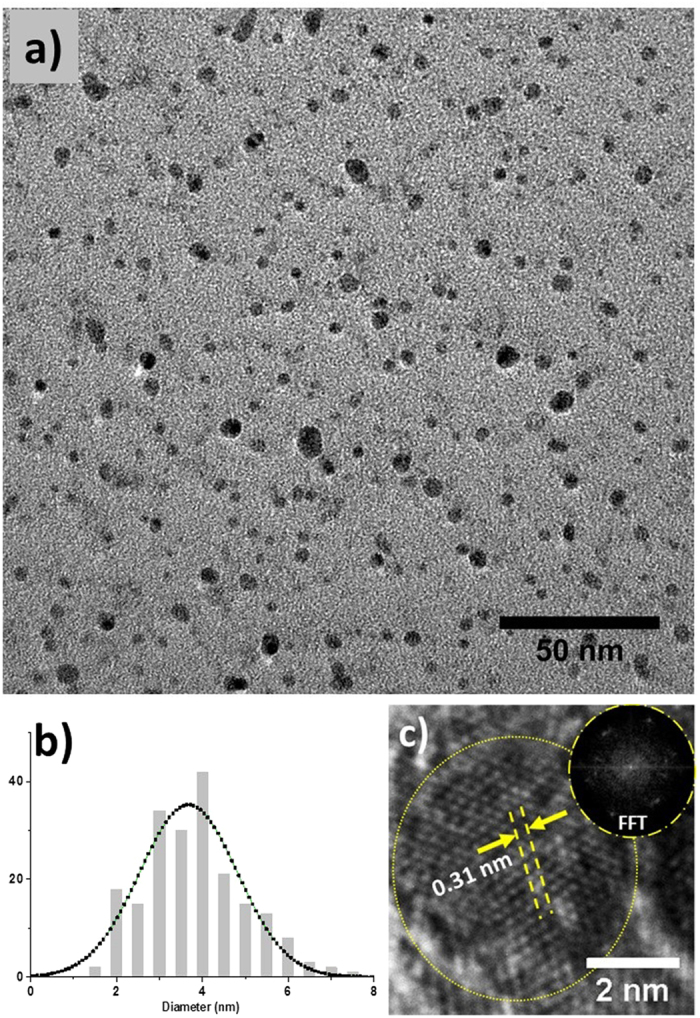
(**a**) Transmission electron microscopy (TEM) image of silicon nanocrystals (SiNCs). (**b**) Histogram of diameter of the particle measured from the figure (**a**). (**c**) High resolution TEM of SiNCs exhibiting typical fringes of crystalline structure; the inset reports the corresponding fast Fourier transform (FFT).

SiNCs are prepared by electrochemical etching where a silicon wafer (p–type boron doped, 0.1 Ω cm, thickness 0.5 mm) was used^12^. SiNCs were collected as powder by subsequent mechanical pulverization. Figure 1 shows transmission electron microscope (TEM) images of unprocessed SiNCs. Samples for TEM analysis consisted of colloidal samples which were first produced by dispersing SiNCs powder in ethanol, then drop-casted and dried on TEM grids. Figure 1a shows a low magnification image of the SiNCs. In order to calculate the size distribution of SiNCs, the diameter of ~200 NCs was measured from low magnification image. Figure 1b reports the size distribution with an average particle diameter of 3.6 ± 1.8 nm. Figure 1c displays a high magnification image showing the crystalline nature of the NCs. The measured d-spacing in Fig. 1c is 0.31 nm, which corresponds to (111) orientation of the cubic face-centered silicon phase with lattice constant of 0.543 nm. The Fast Fourier transformation (FFT) displayed as inset in Fig. 1c shows the symmetries that correspond to the different crystalline planes of the particle under analysis, further confirming its crystalline character. In addition chemical composition and surface characteristics have been extensively studied in our previous reports which included X–ray photoelectron spectroscopy (XPS)^13^ and Fourier transform spectroscopy (FTIR)^4^,^8^,^12^,^14^,^15^.

The studies have been consistent and confirm that these SiNCs are hydrogen–terminated for the most part, with possible dangling bonds, silicon dimers and with a degree of oxidation that depends on the storage conditions. Also the SiNCs are expected to present strong quantum confinement features as their diameter was shown to be between 2–5 nm.

For the work reported here, two different types of colloidal samples were produced and compared: (a) unprocessed SiNCs which were then aged in ethanol for 1 h and (b) SiNCs processed by RF microplasma in ethanol for 30 min which also aged for 30 min following to processing. The concentration of the SiNCs was 1 mg mL^−1^ and no filtration process was applied. However, in order to remove the largest aggregates, the dispersion was left to settle for 10 min and the supernatant part has been used to produce a 10 mL sample of the ethanol-SiNCs colloid.

**Figure 2:**
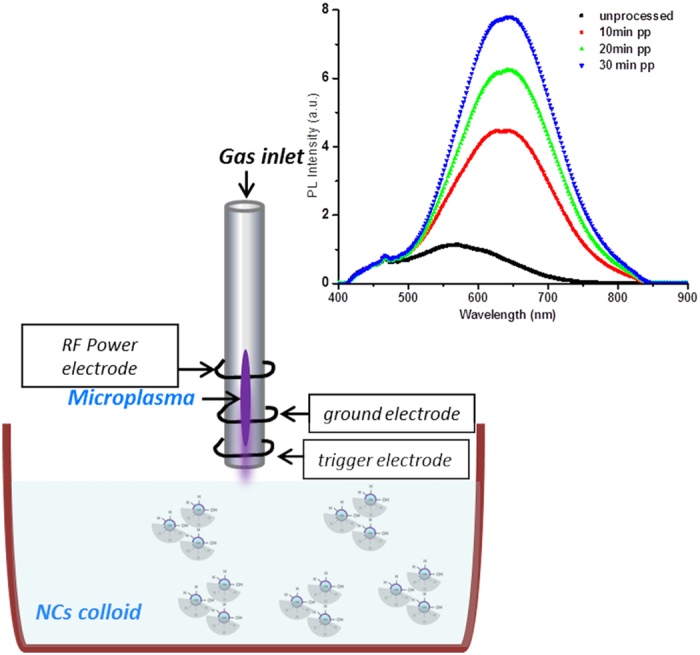
Schematic of the radio frequency (RF) microplasma set-up. The inset reports the photoluminescence improvement after RF-microplasma processing of silicon nanocrystals (SiNCs) in ethanol; ‘pp’ stands for ‘plasma processed’.

Figure 2 shows the RF–microplasma set–up that is being used for surface engineering. This set–up was described in our previous work^4^,^8^. Briefly, the RF–microplasma was generated between the RF electrode and the ground electrode within a quartz capillary; a trigger electrode placed near the ground electrode was used to facilitate plasma ignition at reduced power. Pure helium gas was flown inside the quartz capillary at the rate of 250 sccm. The applied power at the power supply was kept at 60 W (450 MHz) and the reflected power was around 5 W. The distance between the end of the quartz capillary and the surface of the colloid was initially adjusted at about 2 mm, however it was increased up to 2.5 mm over time as ethanol evaporated during processing. Also in this case we adjusted the distance between capillary and the surface of the colloid every 10 min processing. The temperature-dependent measurements were carried out with a Horiba Jobin Yvon Spectrofluorometer (Fluoromax-4) and a cryogenic chamber (i.e. a closed cryostat) built in the spectrofluometer. The excitation wavelength was at 350 nm from a Xe lamp and through a monochromator. The power density was estimated to be 120 mW cm^−2^ in order to assure single exciton per nanocrystal. A 400 nm cut-off filter was used to prevent the excitation signal reaching the detector. Data were corrected on the basis of the system spectral response function. The inset of Fig. 1 shows the enhancement of the PL intensity after surface engineering of SiNCs by RF microplasma in ethanol.

In order to prepare the samples for the QY measurements, the two colloids were drop–casted on silicon wafers and dried inside a fume hood for 2 hours. The dried SiNCs on silicon wafers were then placed inside an integrating sphere (Horiba Scientific). The integrating sphere is connected to an ultraviolet (UV) laser diode and a Horiba Jobin Yvon spectroflurometer through a fibre optic cable. The method followed for measuring QY of NCs was presented by De Mello *et al*.^16^ and Mangolini *et al*.^17^. For comparison, QY measurements also include a sample of porous silicon that did not undergo any mechanical pulverization and was therefore exposed to air for 2 hours.

## Results

The PL properties of SiNCs microplasma processed in ethanol have been previously studied and compared with unprocessed SiNCs^8^,^18^. although in that case a direct–current (DC) microplasma was used, DC and RF plasma–liquid systems have been already compared showing only differences in the rate of SiNCs surface engineering^12^.

Briefly, the PL intensity of unprocessed SiNCs aged in ethanol degrades over time and the peak wavelength progressively blue–shifts; in contrast, RF processed SiNCs in ethanol have enhanced PL and exhibit a pronounced red–shift (e.g. see inset of Fig. 2). Furthermore, the PL of surface engineered SiNCs remains stable over days after microplasma processing as reported earlier^8^,^12^.

Here we report and compare as–prepared unprocessed SiNCs and RF processed SiNCs in terms of their QY and temperature–dependent PL properties. Absolute PL QY measurements allow us to evaluate changes in PL from SiNCs and do not depend on the concentration of SiNCs in solution^17^.

**Figure 3:**
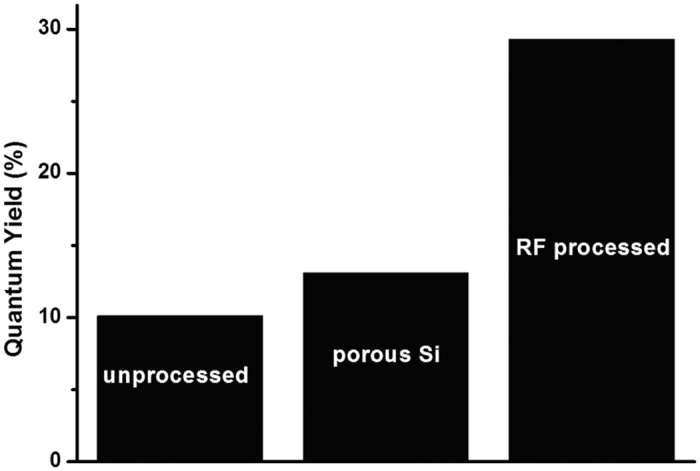
Quantum yield (QY) results show the improvement of the plasma processed sample in ethanol (RF processed) compared to the silicon nanocrystals aged in ethanol (unprocessed) and to the QY of dry porous silicon (porous Si).

Figure 3 shows the QY measurements of the unprocessed and processed SiNCs. For comparison, QY from porous silicon is also reported (13.1%). RF-microplasma processing of SiNCs in ethanol shows an improvement of ~3 folds compared to unprocessed SiNCs. The higher QY of porous silicon compared with the SiNCs powder in ethanol is probably due to the limited oxidation of SiNCs when in the porous structure as many SiNCs in porous silicon are not exposed to water vapor and air because protected by other surrounding SiNCs.

The localized surface states can be affected by disordered potential at the surface induced by different surface terminations, defects or dangling bonds, surface roughness and variation in surface stoichiometry^19^. Therefore it is highly possible that the density of states is changed after microplasma processing. If the surface is less defective then surface states provide radiative recombination paths. This phenomenon happens in plasma–processed samples, where the SiNCs surfaces are more emissive compared to unprocessed SiNCs. Therefore, the more efficient light emission from the SiNCs after microplasma processing shows that the surface terminations and bonding arrangements may affect the recombination process^4^,^12^. It has been already reported that the relaxation in the SiNCs is likely to happen through surface states for oxygen–passivated SiNCs. However, relaxation mechanisms of carriers in SiNCs are generally complex phenomena and the result of the interplay of surface and defects states as well as indirect behavior and quantum confinement. In order to understand this complex phenomena, temperature–dependent PL has been very often applied^19^,^20^,^21^,^22^,^23^. Firstly, we should note that PL emission spectra from SiNCs show clearly and consistently three major PL bands due to transitions that may originate from different energy states such as core energy states, radiative surface states, Si–oxidized surface states, defect states etc.

**Figure 4:**
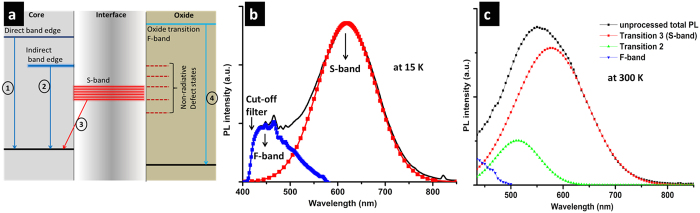
(**a**) Diagram depicting possible transitions in silicon nanocrystals (SiNCs); (**b**) an example of photoluminescence (PL) from SiNCs deconvoluted in two components (PL at 15 K of RF processed SiNCs); (**c**) example of PL from SINCs deconvoluted in three components (PL at 300 K of unprocessed SiNCs).

Probable transitions in SiNCs with oxygen–based surface states are depicted in Fig. 4a: transitions across the direct bandgap (1), transitions due to recombination across the indirect–bandgap (2), transitions from surface states (3) often referred to as “S–band” due to the slow decay time^19^,^23^,^24^ and transitions within the oxide (4). The origin of the S–band (transition 3 centered at ~600 nm) is still debated and many authors explain its origin with quantum confinement models whereas several other authors propose that the broad luminescence originates from carrier trapping and recombination at the surface oxide bonds which produce stable states in the bandgap^25^,^26^. Sychugov *et al*.^22^,^27^ found that the PL from SiNCs has a narrower PL line width compared to what is expected from thermal broadening at low temperature; therefore, such band could be ascribed to quantum confinement and originating from atomic–like energy states of SiNCs. However, it has been found that S–state PL emission can originate from surface states as well^28^. Therefore the assignment of this S–band is still very controversial. Transition 2 emission from the indirect bandgap is size-dependent and is clearly affected by surface states, especially for strongly confined systems; therefore the corresponding peak wavelength can drastically vary for different samples, from less than 400 nm up to above 600 nm^29^,^30^. The blue emission corresponding to transition 4 is less studied and it has been interpreted as due to the structural defects in the oxide shell^29^. Emission due to structural defects in the oxide shell, generally observed in the 400–500 nm range, exhibit fast decay times and for this reason has been referred to as F-band^31^,^32^,^33^. More recently however, for large SiNCs with radii larger than the Bohr radius or for SiNCs with carbon-based or mixed carbon/oxygen terminations, fast decay times have been also observed for emissions above 500 nm^34^,^35^,^36^. However, these do not necessarily correspond to the same “F-band” initially referred to in the literature and attributed to oxide-related emission. With regard to emission from direct bandgap transitions (transition 1), this can be observed only under specific high flux excitation conditions^31^,^37^. More specifically, emission from transition 1 is not expected in our measurements because at our excitation flux, fast electron cooling and fast electron trapping at the oxygen-related defects are dominant^37^. Emission from the direct bandgap transition 1 and F-band emission due to oxide defects can in some cases overlap and would require special attention to discern the different contributions^29^,^31^,^37^. Overlap is observed for instance when the diameter of the NCs is greater than 4 nm^37^. However, this is not the case here because the F-band wavelength range is insensitive to the NC size and therefore still expected within the 400–500 nm range while emission from direct bandgap transitions (transition 1) is expected to be above ~525 nm for 3.7 nm diameter NCs^37^. Interference between the F-band emission and that from the indirect bandgap (transition 2) can also occur as the indirect bandgap is expected to widen with decreasing NC size; in particular transition 2 emission is expected within the 400–500 nm range if the NCs diameter is below 3 nm^38^, which is not the case here.

The transitions in Fig. 4a can all contribute to the PL emission, however their corresponding relative contribution to the PL emission strongly depend on a range of factors (e.g. nanocrystal size, type and “quality” of the interface, temperature etc.) so that PL measurements produce different spectra depending on the SiNCs characteristics. Figure 4a also shows states at the interface which are non–radiative and therefore contribute to quench the PL emission.

As an example, Fig. 4b shows the typical PL spectrum at 15 K of SiNCs after RF microplasma processing where we have assigned the contribution of transition 3 (S-band) and transition 4 (F-band) on the basis of our earlier discussion; we also note that we use the terms “S-band” and “F-band” according to the terminology used in the literature to refer to transition 3 and transition 4, respectively. Transition 1 and transition 2 are believed to have a negligible contribution to the PL emission in this case (see below for the discussion). Another example is represented in Fig. 4c (PL at 300 K of SiNCs aged in ethanol, i.e. unprocessed sample), where in this case also the contribution of transitions across the indirect bandgap can be observed (see below for the discussion).

**Figure 5:**
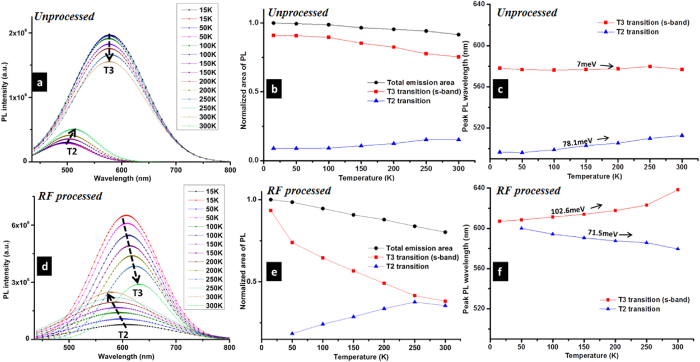
(**a**–**c**) Silicon nanocrystals (SiNCs) aged in ethanol; (**d**–**f**) Radio-frequency (RF) microplasma–processed SiNCs; (**a**,**d**) deconvoluted Gaussian fitted curves to corresponding SiNCs photoluminescence (PL) spectra corresponding to transition 2 (T2) and transition 3 (T3); (**b**,**e**) areas of the fitted Gaussian curves as temperature is varied; (**c**,**f**) wavelength peak shift of the fitted Gaussian curves.

In order to understand the temperature–dependent PL behaviour and to assess the contribution of the different transitions, we have fitted with Gaussian curves all the PL emission spectra (for temperatures from 15 K to 300 K) of both samples, i.e. unprocessed (SiNCs aged in ethanol) and RF processed SiNCs. Transition 3 (S–band) and transition 2 were simultaneously fitted (with the corresponding peaks “forced” to vary within 480–850 nm) while the F–band (transition 4) was then obtained by difference with the original experimental data as the F–band would not affect the fitting process being in the lower wavelength range. Emission from transition 1 was neglected in the fitting procedure for the reasons previously outlined. This procedure allowed us to quantify the contribution of the different transitions to the PL emission for the two samples and at the different temperatures (Fig. 5). Figure 5 therefore reports the fitted Gaussian curves (Fig. 5a,d), the trends of the areas of the fitted Gaussian curves (Fig. 5b,e) and the corresponding changes in peak wavelengths (Fig. 5c,f) for transition 3 (S–band) and transition 2 (across the indirect bandgap) with varying temperature and for both unprocessed SiNCs (Fig. 5a–c) and RF processed SiNCs (Fig. 5d–f). Figure 5b,e have been normalized to the corresponding values of the total area at 15 K.

For both samples, it has been observed that the S–band PL intensity (red square in Fig. 5b,e) decreased when the temperature was increased from 15 K to 300 K. The decreasing trend of the overall PL intensity was observed for SiNCs that were capped by a monolayer of hydrocarbon molecules attached to SiNCs via Si–C bonding^19^. At low temperature the radiative recombination process dominates.

When temperature increases, PL intensity starts to decrease as the non–radiative process also starts to contribute in the relaxation process (see non–radiative defect states in Fig. 3a). Kanemitsu *et al*.^39^ show similar dependence which is ascribed to thermally activated tunnelling effects. Thermally activated tunnelling effect was first proposed by Vial *et al*.^40^, where they proposed that surface carriers may tunnel from a well passivated site to less passivated sites where non–radiative recombination occurs. This mechanism has been further investigated by Suemoto *et al*.^41^ and an assumption was made that the escape process involves tunnelling and thermal activation over a barrier. At the same time carriers diffusion to the surface states from the core would be increased by increasing temperature. However, experimental results from various groups^39^,^40^,^41^,^42^ show that the effect of temperature is more efficient in activating non–radiative processes than enhancing diffusion to the S–states. We therefore believe that all these mechanisms are in part contributing to decreasing PL intensity with increasing temperature for both samples.

In the unprocessed SiNCs, as the temperature is increased, the carriers that populate the S–band are lost to both non–radiative interface states as well as to states of the indirect bandgap; this is confirmed by the increasing intensity for transition 2 and by the overall decrease in PL intensity (blue triangle and black circle in Fig. 5b, respectively). States in the indirect band are also expected to be filled by the direct band states as the temperature increases, contributing further to the increasing transition 2 PL emission. However we should note that transfer between the direct and indirect states happens with phonon emission and therefore the contribution of increasing temperature is minimal as seen in the weakly temperature-dependence of transition 2 in Fig. 5b. The S–band carriers do not seem to transfer to F–band states as transition 4 remains unchanged for this sample (see further below). Further confirmation comes from the analysis of the wavelength position (Fig. 5c), which shows a small red–shift (~78.1 meV) of the indirect transition 2 due to the population in the lowest indirect bandgap states; population of the lower states within the indirect bandgap may be due to either carrier transfer from the S–band states or due to enhanced cascading within the indirect band^39^. Of course the increasing intensity (Fig. 5b) and red-shift of transition 2 (Fig. 5c) is also consistent with a combination of thermal expansion and electron-phonon interactions as it would be for bulk silicon^21^ however, likely, these are not the only factor contributing to the temperature-dependence observed here. The presence of other contributing factors is also confirmed by the 78.1 meV red-shift which is greater than the 47 meV expected for bulk silicon, the difference attributed for instance to tensile strain^31^. For the RF processed SiNCs, S–band states are also depopulated in favour of non–radiative states as well as indirect bandgap states, due to the increasing temperature. However, S–band states in this case are also subject to effective carrier transfer to F–band states where the corresponding transition 4 is seen to increase (see further below). This can be justified by an overall improved passivation of the RF–processed SiNCs and supported by transition 3 (S–band) drastic red–shift 102.6 meV which indicates a dramatic de–population of its higher states. Interestingly, transition 2 through the indirect bandgap is blue–shifted in this case, which is evidence of the contribution of various population/de-population processes that differ from the unprocessed SiNCs and from bulk silicon^21^. The blue shift here can be due to a higher population of the corresponding higher states, possibly from the direct bandgap as it would be expected for higher temperatures.

**Figure 6:**
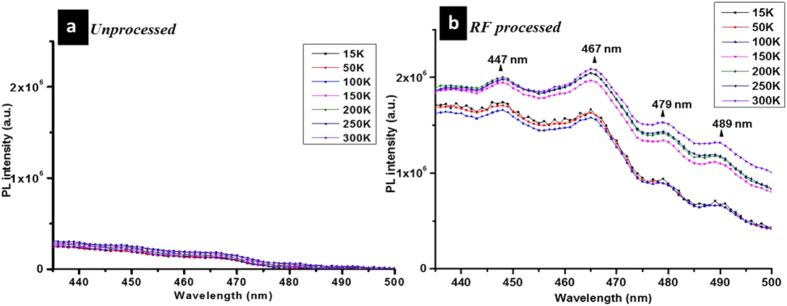
F–band photoluminescence recorded from (**a**) unprocessed silicon nanocrystals “aged” (SiNCs) and (**b**) processed SiNCs in ethanol for different temperatures.

For the F–Band (Fig. 6), the presence of vibronic peaks found in the RF processed SiNCs (Fig. 6b) that do not vary with temperature, indicates an involvement of surface molecular terminations: we have found vibronic peaks at 447 nm, 467 nm, 479 nm and 489 nm (Fig. 6b). The F-band emission in the RF processed samples (Fig. 6b) is weakly dependent on temperature; the overall intensity is increasing with increasing temperature, especially above 100 K, which is consistent with defect-related emission of silicon oxide^31^. In the unprocessed sample, we cannot observe any of these vibronic peaks (Fig. 6a); however, their absence is due to the SiNCs ageing in ethanol (>1 h) and oxide growth.

**Figure 7:**
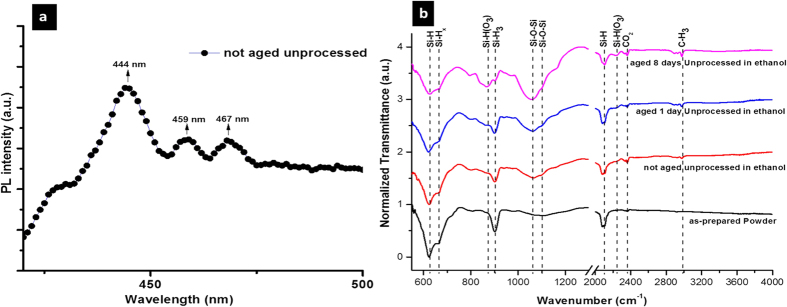
(**a**) Room temperature photoluminescence emission for the F-band recorded from freshly prepared silicon nanocrystals (SiNCs) in ethanol. (**b**) Fourier transform infrared transmission signal of dry SiNCs powder and aging of unprocessed SiNCs in ethanol.

In order to verify the presence of oxide–related molecular peaks in the unprocessed sample during the very initial stages of oxidation we have prepared a sample of SiNCs/ethanol colloid and measured immediately (≪1 h) the corresponding PL (see “not aged unprocessed” sample in Fig. 7a); here we can notice that vibronic peaks are indeed present. In this fresh sample (“not aged unprocessed” in Fig. 7a), vibronic peaks at 444 nm, 459 nm and 467 nm are clearly visible. The emission around 444 nm (~2.8 eV) is associated with a defect pair consisting of Si(O_2_) and Si˙˙ of the adjacent germinal Si–OH groups^14^,^43^. The 459 nm (2.7 eV) vibronic peak is attributed to a blue defect–related emission band in amorphous SiO_2_ at 2.7 eV, ascribed to a Si atom with only two neighbouring oxygen^44^,^45^. The 467 nm peak can be attributed to the finite potential barrier at Si/SiO_2_ interface or Si/Si–O–R interface^46^,^47^.

After ageing for 1 h (Fig. 6a), the oxidation process is expected to have passivated dangling bonds, so that both the 444 nm and 459 nm have disappeared. However, overall the PL emission is degraded due to the formation of strained bonds, as a result of low–temperature oxide growth, which act as sites for non–radiative transitions. This is supported by the corresponding FTIR analysis in Fig. 7b, which shows decreasing absorption due to Si-H terminations and increasing oxide-related absorption over a few days of SiNCs storage in ethanol. FTIR suggests that the molecular vibronic peaks can be observed only in correspondence of a limited degree of oxidation.

Microplasma processing is expected to fully passivate the SiNCs surface with Si–O–R terminations, providing long–term stability of the optical properties of the SiNCs^3^,^8^. Improved passivation is therefore corroborated by the absence of the defect–related peak at 444 nm in RF processed SiNCs (Fig. 6b).

However, the vibronic peak at 467 nm (2.66 eV) remains and it is enhanced in this sample as it is originating from Si–O–R plasma–induced passivation. Surface engineering of the SiNCs lowers the bond potential energy at the Si–O interface which provides a strong carriers confinement inside the Si-core resulting in higher PL emission^48^. Therefore the 467 nm vibronic peak indicates very strong interaction between the exciton wavefunction and surface molecular termination. The other vibronic peaks in the RF processed sample (Fig. 6b) located at the 447 nm, 479 nm and 489 nm, are minor peaks but their origin remains unknown at this time.

Overall in Fig. 6, it can be observed that the F–band PL intensity of the unprocessed SiNCs is generally very low and with an almost negligible increasing trend with increasing temperature. F–band PL emission of processed SiNCs is stronger in intensity and with a stronger temperature dependence, which supports the possibility of carriers thermal activation from the S-band as previously suggested.

## Conclusions

RF microplasma processing of SiNCs suppresses the surface defect states and induces surface states that enhance radiative recombination as a whole. As a result microplasma–processed SiNCs exhibit enhanced PL and QY compared to the as-prepared SiNCs. The results show that surface–engineered SiNCs at low–temperature exhibit a PL emission that is mainly due transitions from the S–band, which may suggest that carrier diffusion to these surface states (from both the direct or indirect band–edges) is very effective even at low temperature. At higher temperatures, carriers have sufficient energy to redistribute and contribute to all radiative transitions and only in minor part through non-radiative defect states as these are greatly reduced by the microplasma process. SiNCs that have not undergone the surface treatment present again a strong PL emission originating from the S–band; however the QY is much lower than processed SiNCs because in this case carriers can diffuse from indirect/direct band–edges to non–radiative states in addition to the S–band. The effect of the temperature on unprocessed SiNCs is weaker because of effective non–radiative dynamics even at low temperatures.

Microplasma processing confirms to be a viable surface treatment for SiNCs extendable to a wide range of other materials. The plasma-induced chemistry and characteristics produced at the surface of SiNCs are advantageous and unique. Importantly, temperature-dependent results produced here have shown to provide a valuable contribution to the understanding of SiNCs and to discern transition dynamics arising from different carrier populations.

## Additional Information

**How to cite this article**: Mitra, S. *et al*. Temperature-dependent photoluminescence of surface-engineered silicon nanocrystals. *Sci. Rep*. **6**, 27727; doi: 10.1038/srep27727 (2016).
